# The MRGPR family of receptors in immunity

**DOI:** 10.1016/j.immuni.2023.12.012

**Published:** 2024-01-09

**Authors:** Naina Gour, Xinzhong Dong

**Affiliations:** 1 Solomon H. Snyder Department of Neuroscience, Johns Hopkins School of Medicine, Baltimore, MD; 2 Howard Hughes Medical Institute, Chevy Chase, MD, USA

## Abstract

The discovery of Mas-related G protein-coupled receptors (Mrgprs) has opened a compelling chapter in our understanding of immunity and sensory biology. This family of receptors, with their unique expression and diverse ligands, have emerged as key players in inflammatory states and hold the potential to alleviate human diseases. This review will focus on the members of this receptor family expressed on immune cells and how they govern immune and neuro-immune pathways underlying various physiological and pathological states. Immune cell-specific Mrgprs have been shown to control a variety of manifestations, including adverse drug reactions, inflammatory conditions, bacterial immunity, and the sensing of environmental exposures like allergens and irritants.

## Introduction

The Mas-related G protein-coupled receptor (MRGPR) family of orphan G protein-coupled receptors (GPCRs) are a cornerstone in somatosensation.^1^ However, their role exceeds the domains of itch and pain, as these GPCRs substantially impact immune cell function. Mrgprs are expressed on various immune cell types, including mast cells, dendritic cells and neutrophils, highlighting their capacity to regulate a variety of immune responses. MRGPR stimulation can trigger different immune processes, leading to the release of pro-inflammatory cytokines, chemotaxis of immune cells, and modulation of immune cell activation states. One of the main identified mechanisms for MRGPRs is facilitating the crosstalk between sensory nerves and immune cells. Neuropeptides released from sensory nerves can activate MRGPRs on immune cells, leading to their recruitment and activation in response to allergens, irritants, and infections. Clinical trials of drugs inhibiting MRGPRs to treat and manage immune-related disorders are already underway, suggesting the possibility that several other MRGPRs with unknown immune-modulating functions could also serve as potential therapeutic targets.

### Brief Historical Perspective

**Figure 1:**
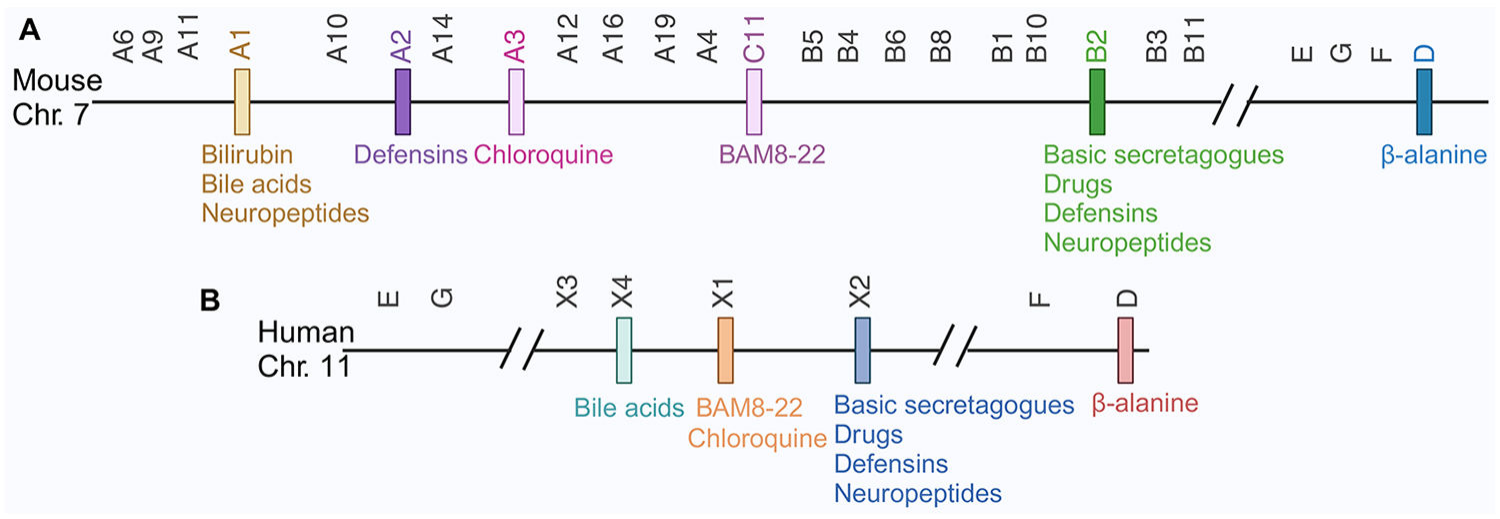
Structure of *MRGPR* receptor family in mice (A) and humans (B) and their ligands. Each bar represents a *Mrgpr* gene.

In 2001, via a subtractive cloning screen using RNA from dorsal root ganglion (DRG) neurons, Dong, Anderson, and colleagues discovered a previously unknown GPCR gene family.^2^ Following these findings, another group discovered these family members using rat neuronal tissue.^3^ As the genes in this family showed the highest homology (approximately 35%) to the MAS1 proto-oncogene, they were designated as the MAS-Related G-Protein Coupled Receptor (MRGPR). Based on sequence similarities, 3 subfamilies are clustered into Mrgpra, Mrgprb and Mrgprc, containing 50 members, of which only 22 have intact open reading frames (ORFs), and the rest are pseudogenes (Fig. 1A). Four human Mrgpr genes showed close homology (approximately 50%) to these 22 mouse Mrgprs. However, without apparent sequence homology needed to confirm the ortholog pair, these 4 human MRGPRs were initially coined as members of the ‘X’ cluster: MRGPRX1, MRGPRX2, MRGPRX3, and MRGPRX4.^4^ Further, 4 other Mrgprs with well-defined sequence homology in both mice and humans were identified, specifically, Mrgprd, Mrgpre, Mrgprf, and Mrgprg. In sum, 26 murine and 8 human MRGPRs with intact coding sequences constitute this receptor family (Fig. 1B).

The Mrgpr family members show high structural similarity, having short (3-21 amino acid) N-termini and relatively conserved transmembrane and intracellular domains. The most variation among these family members resides in the extracellular environment, which imparts unique ligand binding potential.

### The mast cell-MRGPRX2 axis in skin infection and inflammation

The first report of extra-neuronal expression of MRGPRs came in 2006 when Tatemoto et al. demonstrated that human mast cells express MRGPRX2.^5^ This discovery has led to a wealth of new research re-evaluating our understanding of what drives mast cell activation. Classically, mast cells are most known for their role in immunoglobulin E (IgE)-mediated allergic manifestations. In allergen-sensitized individuals, crosslinking of the high-affinity IgE receptor (FcεR1) by antigen-IgE complexes on mast cells drives degranulation, leading to the release of pre-formed mediators such as histamine, arachidonic acid metabolites, and cytokines.^6^ Although mast cells are known for their pre-formed content, they also synthesize lipid mediators and cytokines in response to IgE crosslinking.^7^ The secretory output of activated mast cells drives the hallmark features of type I hypersensitivity (hives, edema, bronchospasm, itching, etc). However, it had been well-established that mast cells can be triggered in an IgE-independent fashion, culminating in an inflammatory response similar to IgE-dependent activation. Before the discovery of IgE in 1967, and starting at least in the 1950s, reports were studying the effects of various mediators, including neuropeptides, on human mast cells and in later experiments beginning in the 1970s, had demonstrated that neuropeptides like substance P (SP) could drive human skin mast cell degranulation.^8-10^ Today, we know that these IgE-independent endogenous and exogenous mast cell activators, most of which are positively charged and are also collectively called basic secretagogues, form a diverse collection of synthetic and natural molecules like compound 48/80, FDA-approved drugs, neuropeptides, venoms, and toxins, now known to trigger mast cells via MRGPRX2.

In 2015, it was reported that Mrgprb2 was the mouse ortholog of human MRGPRX2 ^11^, with similarly restricted expression to mast cells. Thus, MRGPRX2 and Mrgprb2 became a *bona fide* marker of mast cells. However, their expression is not equally found across different types of mast cells, highlighting the developmental dichotomy to the genesis of mast cells. The long-held belief that circulating bone marrow (BM) derived precursors give rise to tissue mast cells is now being replaced with the knowledge that in mice and humans, mast cells can originate from hematopoietic stem cells from the bone marrow or the yolk sac.^12, 13^ Mast cells with embryonic origins (i.e. yolk sac) tend to be enriched for granules containing tryptase, chymase, and carboxypeptidase (MC(TC)). They are found in connective tissues, fat, and sub-mucosa of the skin, in proximity to blood and lymphatic vessels, and neurons.^14^ MRGPRX2 is highly expressed in fat and skin mast cells^15^, and like in humans, Mrgprb2 is also majorly expressed by mouse connective tissue-associated MC(TC).^16^ Unlike yolk-sac-derived mast cells, mast cells that develop from the bone marrow are predominantly found in respiratory and gastrointestinal mucosal tissues and contain tryptase-loaded granules (MC(T)). In mice and humans, these express little Mrgprb2 ^16^ or MRGPRX2 ^15^, respectively. In line with the segregated expression of Mrgprb2/MRGPRX2 to connective tissue mast cells, engraftment of human fetal liver CD34^+^ hematopoietic stem cells into humanized mice resulted in the development of human mast cells in various organs where it was observed that only the skin engrafted mast cells expressed MRGPRX2, but not those in the lungs, spleen or liver.^17^ This may suggest the possibility that connective tissue-specific signals may support the expression of MRGPRX2.

Ligand discovery efforts using peptide and chemical screening libraries revealed that both Mrgprb2 and MRGPRX2 could be ligated by hundreds of compounds, including many endogenous secretagogues ^18^, which have been shown to trigger mast cell degranulation. These include substance P (SP), pituitary adenylate cyclase-activating polypeptide (PACAP), somatostatin (SST), cortistatin, the adrenomedullin-derived PAMP12 (also known as PAMP9-20), and anti-microbial peptides.^5, 11, 18^ In addition, Mrgprb2/MRGPRX2 were shown to bind various cationic FDA-approved drugs, including vancomycin, icatibant, ciprofloxacin, as well as several compounds derived from the opioid family like codeine and morphine.^11, 19, 20^ Engagement of this pathway resulted in mast cell degranulation and the development of drug-induced pseudo-allergic drug reaction in mice, suggesting that MRGPRX2 is may be responsible for adverse drug reactions like the red man syndrome characterized by itchy skin eruptions in response to vancomycin.^21^

Mast cells are a versatile component of innate immunity, and they essentially populate all organs. Mast cells are considered one of the most ancient immune cells originating nearly 500 million years ago from primitive test cells of urochordates, which are functionally and morphologically highly similar to mast cells, including their ability to degranulate and respond to compound 48/80 (C48/80).^22^ Like their ancestor test cells, which were thought to provide host protection against infections, Mrgpr-expressing mast cells are likely central to how we have evolved a rapid response to environmental triggers and pathogen exposures. This detection system can react to various host mediators released during infection, as well as molecules of microbial origin. One of the major anti-microbial pathways thought to be elicited during infection is the recognition of host-defense peptides by Mrgprb2/MRGPRX2 on mast cells.

Cationic host-defense peptides also known as anti-microbial peptides (AMPs) are expressed across diverse species ranging from microorganisms to plants, amphibians, and mammals.^23^ These short (>50 amino acids), positively charged (at physiological pH) peptides from vertebrates can be categorized into 2 broad classes, defensins and cathelicidins.^24^ Defensins are broadly subdivided into 3 categories: alpha-defensins, beta (β)-defensin and θ-defensins; among these, beta-defensins are ubiquitous and found in all vertebrates.^24^ LL-37 is the only human cathelicidin.^25^ Both LL-37 ^26^ and β-defensins ^27^ are ligands for MRGPRX2 and induce MRGPRX2-dependent mast cell degranulation.

Given their anatomical distribution at barrier surfaces, mast cells are known to be actively engaged by pathogens, including viruses, bacteria and fungi, where they generally provide protective immunity.^28, 29^ During infection, mast cells can be activated downstream of cell-surface receptors, like toll-like receptors (TLRs) and Fc-receptors (FcRs)^30^, and this leads to the secretion of a wide variety of mediators that are directly anti-microbial such as AMPs.^31^ A major mechanism by which mast cells are thought to mediate anti-microbial response is through increased recruitment and activation of other immune cells like neutrophils.^32^ In a model of localized dermonecrotic infection using an intradermal injection of *S. aureus*, mice with inducible deficiency in mast cells (*Mcpt5*^*Cre*^;*iDTR*) failed to clear bacteria efficiently when compared to mast cell sufficient animals.^33^ Also, injection of the Mrgprb2/MRGPRX2 agonist mastoparan, a peptide isolated from wasp venom, reduced skin bacterial burden, wound size, and scarring in mast cell-sufficient mice, but not when mast cells were depleted. Administration of mastoparan also induced mast cell-dependent recruitment of neutrophils, Langerhans cells and DCs in response to *S. aureus*. In line with this, the inactive mastoparan analog, mastoparan 17, was significantly less effective than mastoparan in reducing lesion size. However, several cationic amphiphilic peptides, such as mastoparan, not only activate mast cells via Mrgprb2/MRGPRX2 but can also kill bacteria directly by binding to and disrupting negatively charged bacterial membranes. Thus, Arifuzamman et al. ^33^ generated mastoparan analogs (Duke Mast F and MP-6I peptides) with uncoupled properties to separate the mast cell degranulating and direct anti-bacterial properties of mastoparan. The Duke Mast F peptide induced mast cell activation but had no anti-bacterial activity and was able to reduce *S. aureus*-induced lesion size. Conversely, the MP-6I peptide had no activity on mast cells but retained its anti-bacterial properties and showed no effect in reducing lesions. Thus, suggesting that sensing of endogenous anti-microbial peptides by Mrgprb2/MRGPRX2 is a key anti-microbial mechanism.

**Figure 2:**
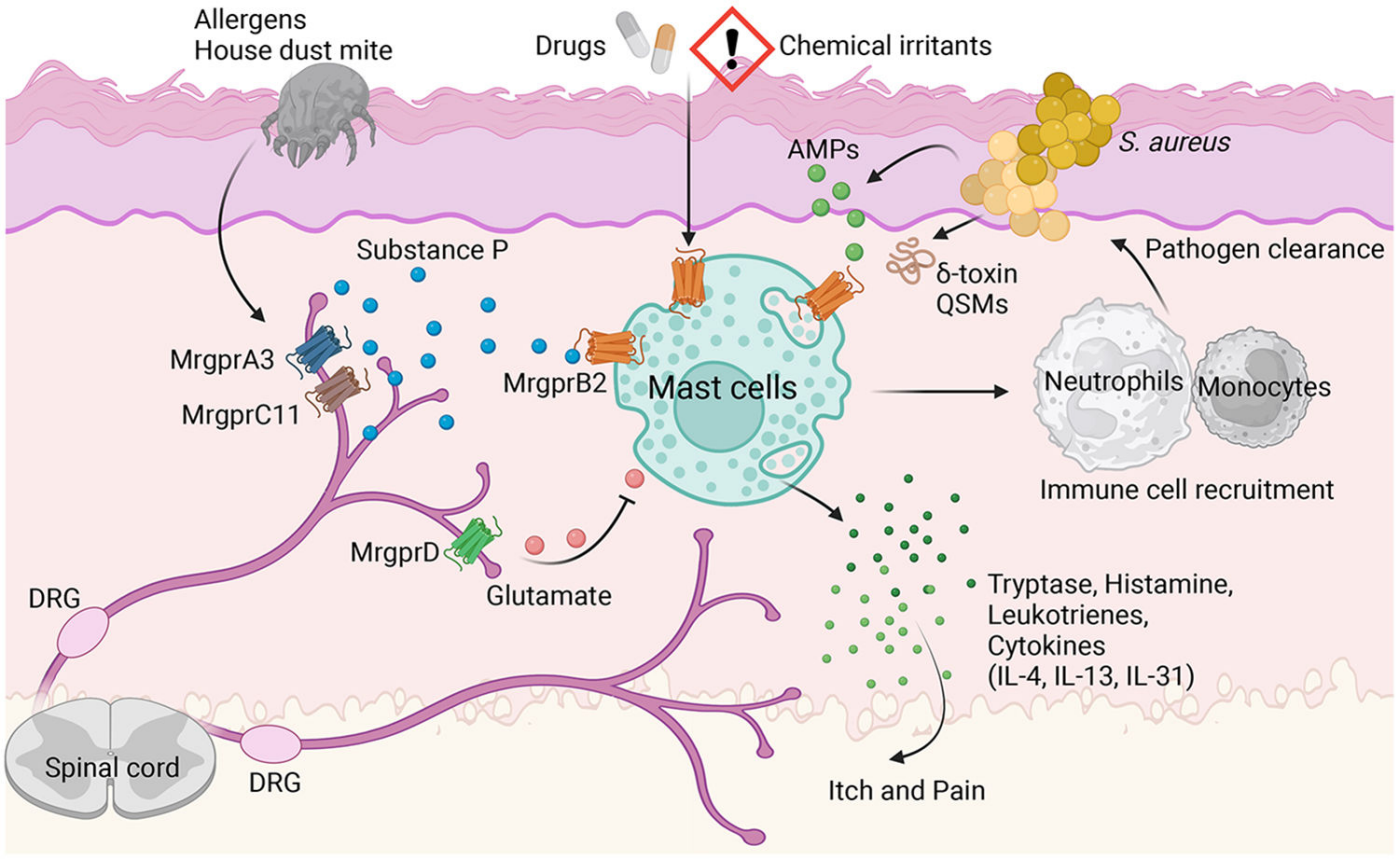
Mrgprb2 on mast cells can be activated by variety of endogenous and exogenous positively charged activators (e.g. neuropeptide substance P, drugs, chemical irritants, AMP, QSM, etc). The activation of Mrgprb2 on mast cells leads to the release of protease, cytokine, chemokines and recruitment of other immune cells. There are also mutual interactions between mast cells and sensory nerves in the skin leading to itch and pain.

A recent study showed that Mrgprb2-mediated recognition of anti-microbial peptides is not the only mechanism by which mast cells protect the host. Bacterial gene expression is dictated by their cellular density in a phenomenon known as quorum sensing (QS). Both gram-negative and gram-positive bacteria synthesize QS molecules (QSM), which serve as messengers for communication within the bacterial colony.^34^ However, these can also be sensed by host immune cells. Various cationic QS peptides from gram-positive bacteria were shown to activate Mrgprb2 and MRGPRX2 and mediate host-pathogen clearance.^35^ Various QSM such as the competence-stimulating peptide (CSP) from *Streptococcus pneumoniae*, Entf from *Enterococcus faecium*, and Streptin-1 from *S. pyogenes* activated mast cells through Mrgprb2 and MRGPRX2, leading to mast cell degranulation. Further, CSP-deficient *Streptococcus pneumoniae* deficient could not activate mast cells. During infection, *Mrgprb2^−/−^* mice had elevated bacterial loads and reduced recruitment of neutrophils compared to controls. These findings are in line with others^33^, showing that an intact mast cell response facilitates the influx of immune cells, including neutrophils. This suggests that in addition to the ability of mast cells to directly produce AMPs ^31^, they coordinate the recruitment of other immune effectors to produce an anti-microbial response. Together, these reports support the role for an Mrgprb2/MRGPRX2-mast cell axis in controlling bacterial infections at barrier surfaces (Fig. 2).

Aberrant microbial activity is also thought to be pathogenic in rosacea, a chronic skin inflammatory condition with multifactorial etiology.^36^ Dysbiotic colonization in rosacea is believed to drive a heightened host defense response encompassing enhanced anti-microbial peptide generation and immune cell recruitment.^37^ Compared to normal skin, patients with rosacea harbor more mast cells.^38^ Consistent with this, studies in murine models have demonstrated the importance of mast cells in rosacea-like skin inflammation induced by intradermal injection of the cathelicidin LL-37. ^38, 39^ Moreover, in skin biopsies of patients with rosacea, more mast cells express MRGPRX2 than in normal subjects.^40^ Furthermore, LL-37-induced rosacea in mice was significantly reduced in mast cell deficient *Kit^W-sh/W-sh^* mice and *Mrgprb2^−/−^* mice.^40^

The Mrgprb2/MRGPRX2-mast cell axis may go beyond responding to bacterial infections and could play a role in the host response to tick bites. A recent study showed that Mrgprb2 and MRGPRX2 can recognize tick (*Ixodes persulcatus)*-derived salivary defensin peptides (IPDef1, IPDef2).^41^ *In vivo*, IPDefs elicited scratching and vascular leakage, and *in vitro* stimulation of mouse peritoneal mast cells with recombinant IPDef induces the release of histamine, serotonin, and tryptase, although it is not clear whether this is dependent on Mrgprb2. Nonetheless, taken together this data is suggestive that the tick bite-mediated itch sensation may be dependent on the Mrgpr-mast cell system.

### Mast cells and MRGPRs: the neuroimmune axis regulating dermatitis and itch

Sterile and non-sterile immunity is no longer the purview of only immune cells. In the last decade, the field of neuroimmunology has contributed to a more holistic view of our responses to infection, inflammation and somatosensation. We now know that neuronal signals are essential to condition an appropriate immune response. In many tissues, like the skin, subsets of immune populations, including mast cells, exist near sensory nerves and are receptive to neural outputs.

Our comprehension of the molecular pathways responsible for translating pruritic stimuli into the sensation of itch is still at an early stage. Itch can be categorized into two main origins: histaminergic and non-histaminergic.^42^ As the primary source of histamine, mast cells are crucial mediators of histaminergic-related acute itch. In sensitized allergic individuals, IgE-complex via crosslinking of high-affinity IgE receptors on mast cells can activate them, leading to histamine release.^7^ Histamine-induced itch involves the H1 and H4 histamine receptors on histaminergic nerves, this activates capsaicin-responsive nociceptors or TRPV1^+^ nociceptors, leading to the release of neuropeptides, like SP and calcitonin gene–related protein (CGRP).^43 44^ This so-called “neurogenic inflammation,” results in vasodilation in the surrounding tissue, plasma extravasation, and mast cell degranulation.^42, 45^ While histamine is an important mediator for both acute and chronic itch, non-histaminergic pathways are major contributors to different types of itch. For example, itch neurons can be activated by a variety of exogenous and endogenous nonhistaminergic pruritogens such as peptides, lipids, cytokines and proteases, etc.^46^ While histamine receptors are present in the 3 main classes of itch-sensing neurons in human and mouse, labeled as non-peptidergic nociceptor 1 (NP1), NP2, NP3 ^47^, Mrgprs have selective expression. Single cell RNA sequencing of mouse DRG sensory neurons revealed that NP1, NP2, NP3 are specifically labeled by *Mrgprd*, *Mrgpra3*, and *somatostatin* (*SST*) expression, respectively.^48^ Consistently, human DRG single cell RNAseq study also demonstrated the existence of these three classes of itch-sensing neurons.^47^ In human DRG, *MRGPRX1* is expressed in both NP1 and NP2 whereas *MRGPRD* is weakly expressed in NP1 and *MRGPRX4* is specifically expressed in NP2 population. Like mice, SST is also a specific marker for human NP3 neurons.^47^

Mrgprb2 is an important mediator of non-histaminergic itch. Importantly, itch triggered by Mrgprb2-activated mast cells drives the release of different pruritogens, which results in the engagement of distinct itch sensory neurons compared to those initiated downstream of anti-IgE complexes. The Mrgprb2 agonist PAMP12 induced Mrgprb2-dependent scratching, and this was accompanied by the release of tryptase, but not histamine.^49^ On the other hand, itch induced by anti-IgE complexes or ovalbumin was not dependent on Mrgprb2 and induced histamine, but not tryptase release. The administration of Mrgprb2 agonists, PAMP12 and C48/80, in mice skin activates sensory neurons that partly overlap with those activated by substances like β-alanine, which triggers Mrgprd, chloroquine, which binds to Mrgpra3 and 5-hydroxytryptamine (5-HT).^49^ However, these neurons do not coincide with those sensitive to histamine and capsaicin. Conversely, neurons activated by anti-IgE complex administration overlap with those responsive to histamine and capsaicin but not those triggered by β-alanine, chloroquine, and 5-HT.^49^ This distinction highlights the complexity of itch pathways and the diversity of sensory neurons involved in different itch sensations. Mrgprb2 was also partially involved in driving itch and immune cell infiltration in different models of acute contact dermatitis (ACD) using dinitrochlorobenzene (DNCB), squaric acetyl dibutyl acid (SADBE) and oxazolone. Specifically, in these models, itch and immune cell influx was reduced by approximately 50% in *Mrgprb2^−/−^* mice as compared to controls.^49^ Itch responses not mediated by Mrgprb2 may be elicited downstream of histamine or other alternative pathways of itch possibly involving basophils, natural killer (NK) cells, IL-31, TSLP, or IL-33.^50-53^ Further, as Mrgprb2 agonists favors the release of tryptase, which is a known activator of protease-activated receptors (PARs), it remains a possibility that Mrgprb2-driven non-histaminergic itch involves PAR-mediated activation of sensory fibers.^54, 55^

Atopic dermatitis (AD) a.k.a. eczema and ACD manifest similar symptoms but have different causes: external vs. internal triggers, respectively.^56^ AD is a heterogeneous, chronic allergic inflammatory disease characterized by rashes, skin lesions and intense itch. While the complete etiology of AD is unknown, a dysregulated type 2 immune response with elevated IgE and barrier dysfunction is thought to underlie its pathogenesis.^57, 58^ Mast cells are elevated in the skin of patients with AD and they are thought to play a role in AD pathogenesis.^59^ Additionally, microbial dysbiosis is now appreciated as a potential driver of AD pathophysiology. Compared to non-AD patients, *Staphylococcus aureus* colonization is 20-30% in healthy individuals but reaches 30-100% in AD patients ^60^, and it can colonize lesional and non-lesional skin. This is thought to be driven in part by compromised barrier integrity. *S. aureus* produces a range of virulence factors, including proteases (aureolysin, serine protease, cysteine protease), phenol soluble modulins (PSMs) and superantigens (staphylococcal enterotoxin A, B, TSST-1) which can disrupt barrier function and engage inflammatory circuits driving AD.^61^

It is thought that dysregulated mast-cell-neuron circuits can drive AD. In a murine model of AD utilizing a mixture of house dust mite (HDM) and staphylococcal enterotoxin B (SEB), depletion of TRPV1^+^ nociceptors using the ultrapotent TRPV1 agonist, resiniferatoxin, attenuated HDM+SEB-induced skin pathology and as well as reducing eosinophil, and neutrophil recruitment.^14^ In this model, HDM is thought to trigger skin sensory neurons directly. Specifically, while SEB itself did not, HDM alone or in combination with SEB induced calcium influx in a capsaicin-sensitive subpopulation of DRG neurons *in vitro* and caused the release of SP in culture. SP is then thought to trigger Mrgprb2 on mast cells, leading to inflammation. Functionally, manifestations of experimental AD were almost entirely dependent on the SP-Mrgprb2 axis. Specifically, SP- or Mrgprb2-deficient animals had a significant reduction in skin pathology, inflammation and IgE production in response to allergen.^14^ Interestingly, HDM-induced activation of cultured DRG neurons is independent of MyD88 or the protease-activated receptor (PAR2) but requires the intrinsic proteolytic activity of HDM.^14^ Specifically, the cysteine, but not serine, protease activity of HDM was necessary for neuronal activation. In line with these findings, *Der p 1*, a cysteine protease and major allergen from house dust mite, was shown to activate MrgprC11, Mrgpra3 and the human ortholog MRGPRX1^62^. This activation was entirely dependent on the protease activity of *Derp 1*. We thus envision an Mrgpr relay involving the direct activation of Mrgprs on sensory neurons, leading to the release of Mrgprb2-activating neuropeptides that induce mast cell degranulation (Fig. 2). On the other hand, the serine protease activity in sources of allergens is important in mediating IL-33 release.^63^ Moreover, endogenous serine proteases like mast cell tryptase and chymase potentiates IL-33 mediated ILC2 response.^64^ Aberrant IL-33 production is central in many allergic manifestations, including AD ^65-67^, where it is thought to play a pathogenic role.^68, 69^ Thus, it is reasonable to foresee that allergens can engage two different mechanisms: 1) via a cysteine protease-dependent nociceptor-mast cells module and 2) through a serine protease-induced IL-33 axis, both converging in driving the development of type 2 immunity in AD.

Lastly, it is important to appreciate the nuances in the murine models utilized to study dermatitis and the role of mast cells among these. Mast cells have been found to be largely dispensable in experimental models of AD using MC903 (calcipotriol, a vitamin D analog) ^70^ and acetone:ether-induced dry skin itch.^65^ In simpatico with these, the sensation of itch in MC903-induced AD and acetone:ether-induced dry skin, was independent of Mrgprb2.^49^ Perhaps, the contribution of mast-cell in AD can be appreciated in less reductive models of skin inflammation and likely better studied in approaches mimicking the more complex nature of the environmental triggers (allergen & microbial interaction) that drive AD in susceptible individuals.

While some mast-cell-neuron interactions can drive skin inflammation, others can establish a regulatory tone in the skin (Fig. 2). A group of skin sensory neurons, distinct from TRPV1^+^, express Mrgprd.^71^ These Mrgprd^+^ neurons could be maintained by Langerhans cells and promote homeostasis.^72^ During irritant-induced dermatitis or *S. aureus*-induced skin infection, the Mrgprd^+^ neurons were shown to protect against aberrant mast cell activation in response to chemical irritants via their glutamate release. Selective ablation of glutamate signaling on mast cells induces a hyperactive state. Administration of the Mrgprd ligand, β-alanine reduced mast cell activation in response to the Mrgprb2 agonist C48/80. In line with this, the depletion of Mrgprd^+^ neurons resulted in heightened SP release and hyperactivation of local mast cells via a Mrgprb2-dependent mechanism.^72^ Skin mast cells from Mrgprd^+^ neurons-depleted mice showed increased *Mrgprb2* transcript and enhanced responses to C48/80. Despite the loss of skin homeostasis, the heightened mast cell activity in mice lacking Mrgprd^+^ neurons lead to improved control of *S. aureus* skin infection.^72^ Therefore, while SP-expressing TRPV1^+^ neurons activate the Mrgprb2-mast cell axis, a separate group of Mrgprd^+^ neurons can secrete glutamate to control this aberrant activation. We can envision that dysregulation of this balance could represent a novel disease pathway in susceptible individuals.

### Mast cells, MRGPRs and pain

Unsurprisingly, mast cells have generated significant attention due to their pivotal role in inciting inflammation in response to diverse triggers. Yet, a compelling revelation is the newfound understanding that mast cells, primarily via Mrgprb2-dependent mechanisms, profoundly influence the sensory perception of cutaneous inflammatory pain. Traditionally, the conventional wisdom held that sensory neurons could elicit pain, in isolation from the participation of other cells. It was postulated that sensory neurons could directly sense bacterial products, such as formylated peptides ^73^ or endotoxin recognized through the TLR4 receptor ^74^ but also independent of TLR4, via the TPRA1 channel ^75^, thereby provoking pain during infections. Infection-induced pain was shown to occur independently of neutrophils and monocytes ^73^, and more largely, the role of these cells in driving pain in other models is also debated ^76, 77^. However, the contribution of mast cells remained to be explored. Emerging evidence has demonstrated the existence of neuro-mast cell mechanisms that underlie inflammation-induced pain, which may also be triggered in response to bacterial products. One model frequently employed to mimic inflammatory pain is the use of Complete Freund's Adjuvant (CFA), a suspension of inactivated mycobacterium. In this context, the work of Green et al. has demonstrated the critical role of Mrgprb2 in driving CFA-induced hyperalgesia.^78^ Furthermore, using a relevant model mimicking postoperative incision pain, it was observed that the depletion of mast cells expressing Mrgprb2 (*Mrgprb2^Cre^ROSA2^iDTR^* mice) resulted in a reduction in post-incision-induced thermal and mechanical hypersensitivity compared to controls. Like with CFA, this attenuation of hypersensitivity and pain was dependent on Mrgprb2, as supported by the diminished response observed in *Mrgprb2^−/−^* mice compared to controls. In addition to pain, the increased infiltration of immune cells, specifically neutrophils and monocytes, observed in the postoperative setting, as opposed to sham-treated animals, was entirely abolished in mice deficient in Mrgprb2. Mechanistically, SP-mediated activation of Mrgprb2, but not through its canonical receptor neurokinin-1 receptor (NK1R), could drive cell infiltration and the elicitation of pain. These observations emphasize the pivotal role of the mast cell-Mrgprb2 axis not only in driving immune responses in the skin but also in orchestrating the accompanying perception of pain.

In addition to SP, PACAP, another neuropeptide ligand for MRGPRX2 ^5^ was shown to mediate migraine pain.^79, 80^ Specifically, using *Mrgprb2^Cre^;ROSA26^tdTomato^* mice, it was shown that mouse meningeal mast cells expressed Mrgprb2. Further, in a migraine model, applying C48/80 and PACAP 1-38 directly onto the dura increased mechanical facial hypersensitivity which was significantly reduced in *Mrgprib2^−/−^* mice.^79^ To test the role of MRGPRX2 specifically in mast cells *in vivo*, transgenic mice harboring MRGPRX2 (*ROSA26^lsl-MRGPRX2^*) were crossed with *Mrgprb2^Cre^* mice, generating *Mrgprb2^MRGPRX2^* animals. In order to circumvent the potentially confounding role of endogenous Mrgprb2 in mast cells, Mrgprb2^MRGPRX2^ mice were crossed to *Mrgprb2^−/−^* mice to generate *Mrgprb2^MRGPRX2^;Mrgprb2−/−* mice. This targeting strategy knocked in MRGPRX2 in approximately 70% of peritoneal and meningeal mast cells. Stimulation of these peritoneal mast cells with PACAP resulted in a calcium response in those cells cultured from *Mrgprb2^MRGPRX2^;Mrgprbr2^−/−^* animals; however this was not seen in control animals. Importantly, *in vivo*, dural application of PACAP resulted in migraine-like pain behavior in MRGPRX2-overpressing mice, significantly more than in control animals. While the precise mechanisms underlying how PACAP activates Mgprb2 and MRGPRX2 on mast cells to induce migraine pain remains an area of investigation, there is a possibility that their degranulation could result in the expansion of meningeal arteries. This assumption is rooted in the observation that PACAP has been demonstrated to induce arterial dilation in rats, and this process appears to be mast-cell dependent.^81^ Notably, efforts aimed at targeting the conventional receptors for SP (NK1R) and PACAP (PAC1R) ^82, 83^, have yielded limited success in mitigating pain, including migraine pain.

In line with the importance of mast cell and Mrgprb2 in pain perception, a recent study utilizing a mouse model of alcohol withdrawal-induced headache showed that MrgprB2 significantly impacts pain perception.^84^ Specifically, while WT and *Mrgprb2^−/−^* mice have similar intake and preference for alcohol over water, following alcohol withdrawal, *Mrgprb2^−/−^* mice had less pain (grimace score). They performed better in physical activity (open-field) tests. Using Pirt-GCaMP3 Ca^2^+ imaging, the authors demonstrated that following alcohol-withdrawal, a group of small-diameter (<20 mm) and medium-diameter (20-25 mm) trigeminal neurons are spontaneously activated. However, this is significantly reduced in *Mrgprb2^−/−^* mice. Ethanol consumption and withdrawal activate the Hypothalamic-Pituitary-Adrenal (HPA) axis. This is thought to lead to enhanced corticotropin-releasing factor (CRF) levels in the plasma and dura mater. CRF can then activate mast cells through MrgprB2, inducing the release of TNFα which could sensitize TRPV1 in sensory neurons and mediate alcohol withdrawal-induced pain.^84^ However, CRF has also been shown to have profound analgesic effects, in part through inducing endogenous opioids such as beta-endorphins.^85^ The role of CRF acting through its canonical receptors (CRFR1 and CRFR2) vs. MrgprB2 seems to be distinct. For clinical targeting, it will be necessary to further understand the interplay and usage of receptor subtypes by CRF in pain perception. Overall, MRGPRX2 may serve as an alternative receptor for these pain-inducing mediators (SP, PACAP, CRF), constituting a new and promising target for managing pain.

In the last decade, our understanding of MRGPR-expressing mast cells in an immuno-pathological context has grown (Fig. 2). However, yolk-sac-derived tissue-resident mast cells, akin to resident macrophages, have homeostatic functions, and the role of Mrgpr's in this context is a domain of novel exploration.^12^

Lastly, while the expression of Mrgprb2/MrgprX2 is thought to be largely restricted to mast cells, a few recent studies also indicate that other cell types, like sensory neurons, eosinophils, and basophils, can express MRGPRX2.^86-88^ The extent to which MRGPRX2 is expressed in these at baseline and during inflammation and its function on these cells remains to be studied.

### Mrgprs in dendritic cells and neutrophils

Our current understanding is that, except for mast-cell exclusive Mrgprb2/MRGPRX2, all members of the Mrgpr family are expressed in DRG sensory neurons. However, new findings are highlighting that several Mrgprs are expressed by immune cells.

Mrgpra1 is the founding member of the Mrgpr family, which was initially described in 2001, where it was shown to be activated by a variety of neuropeptides, including neuropeptide FF (NPFF), SP, and SST.^2^ Mrgpra1 is known to be expressed by murine DRG.^89^ Moreover, using *in vitro* cultured mouse DRG, it has been suggested that SP-elicited itch could be driven by Mrgpra1 ^+^ DRG fibers.^90^ Though, the biology of Mrgpra1 extends beyond neurons. A recent report has shown that it is expressed in skin CD301b^+^ dendritic cells (DCs), promoting allergic inflammation. ^91^ In response to intradermal delivery of the protease papain, skin sensory neurons release SP, which acts on adjacent CD301b^+^ DCs through Mrgpra1 The SP-Mrgpra1 axis promoted the migration of CD301b^+^ DCs to local lymph nodes (LN), as *Mrgpra1^−/−^* CD301b^+^ DCs displayed significantly less migration than control CD301b^+^ DCs. LN recruitment of CD301b^+^ DCs promoted papain-induced Th2 responses and the development of skin allergy.^91^Although papain has been shown to directly activate human mast cells ^92^, papain-induced itch and numbers of CD301b^+^ DCs in the LN remained unaffected in mast cell-deficient (*Kit^W-sh/W-sh^* ) mice.^91^ These data demonstrate that Mrgpra1 could function to enhance type 2 immunity in the skin in response to environmental triggers.

Other members of the Mrgpra subfamily, Mrgpra2a, and Mrgpra2b, are characterized by their expression in DRG neurons^2^ and neutrophils ^93^. While the function of DRG-expressed Mrgpr2a/2b remains to be explored, a recent study demonstrated that β-defensins, secreted by keratinocytes during skin infections, can engage Mrgpr2a/2b on neutrophils to promote anti-bacterial responses.^94^ Mice deficient in keratinocyte-derived defensins or deficient for Mrgpr2a/2b displayed impaired bacterial clearance compared to control animals in a *S. aureus* skin infection model. The stimulation of neutrophils with human beta-defensin-3 (hBD3), or its mouse homolog BD14, led to neutrophil degranulation, which was abrogated in neutrophils deficient for Mrgpr2a/2b (Mrgpra2 DKO). Further, at baseline, the bacterial species composition of skin differed considerably between control animals and mice deficient in either defensins or Mrgpr2a/2b. Given the importance of the microbiome in maintaining skin homeostasis, it is likely that this pathway could participate in the pathobiology of various cutaneous conditions by regulating the skin microbiome. Moreover, as several endogenous cationic antimicrobial peptides^95^ including β-defensins can ligate Mrgprb2/MRGPRX2 on mast cells, Mrgpra2a/2b on neutrophils, as well as other Mrgprs ^95^, we hypothesize that together, this form a united front to microbial aggressors.

### Mrgprs structure and signaling

**Figure 3:**
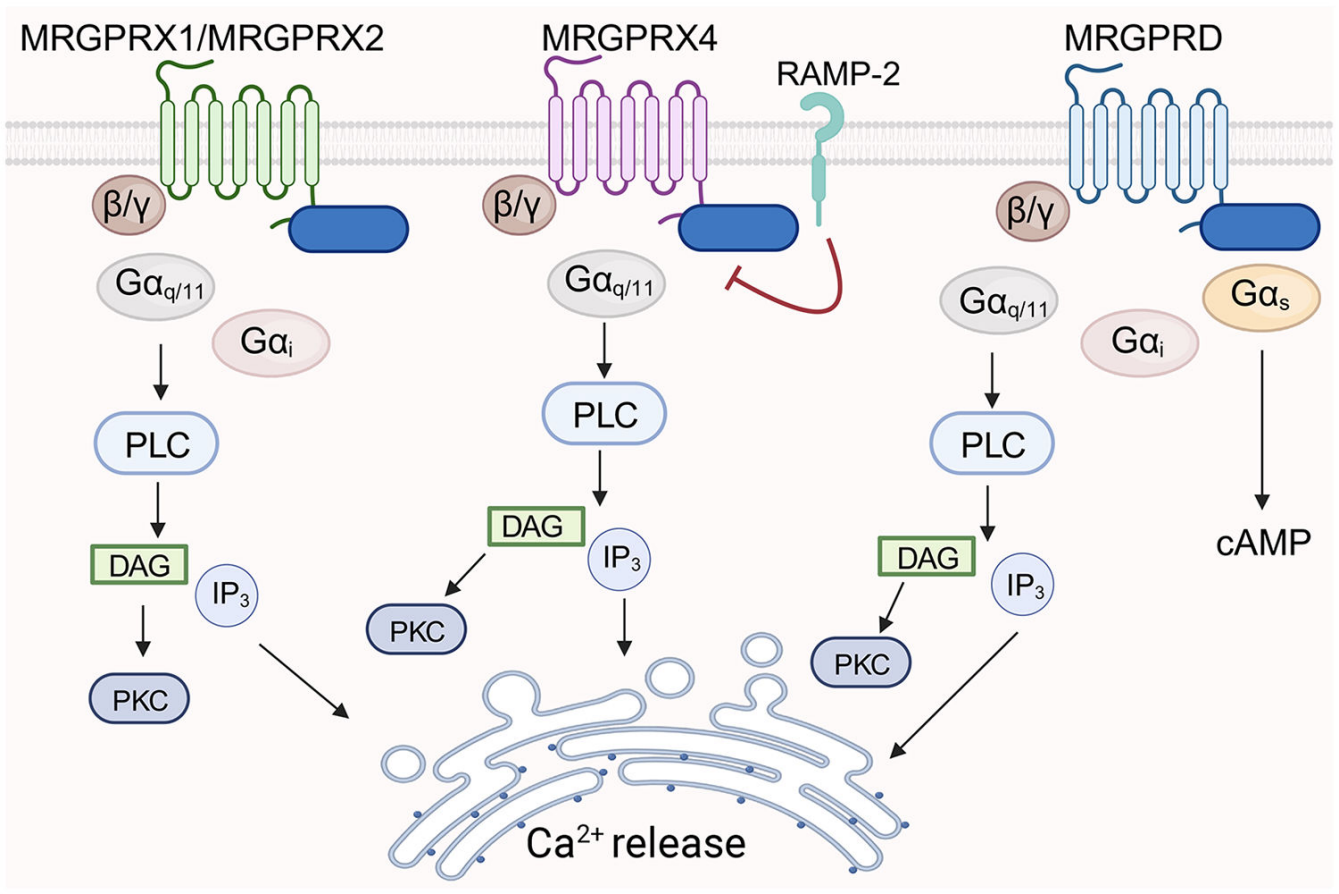
Signaling mediators downstream of MRGPRs. MRGPRX1, MRGPRX2, MRGPRX4 and MRGPRD can mediate Gα_q11_ dependent pathways leading to phospholipase C (PLC) driven calcium responses. MRGPRX1, MRGPRX2 and MRGPRD can additionally engage Gαi and MRGPRD can recruit Gαs that drives cAMP production. In addition to G-proteins, β-arrestin signaling is engaged by MRGPRs and selectively other cell-surface receptor like RAMP-2 can inhibit MRGPRX4 expression and basal or agonist-induced activation.

Canonically, GPCR signaling involves three heterotrimeric G proteins (Gα, Gβ, and Gγ), which associate with the receptor in an unstimulated state. Ligand binding leads to a conformational change in the GPCR, triggering binding of the Gα subunit to GTP, producing a downstream signaling cascade.^96^ The Gα subunit exists in 4 families: Gαs (s-stimulatory), Gαi (i-inhibitory), Gαq11 and Gβq12/13. Mrgprs can utilize all G-proteins (Gαs, Gαi, Gαq) (Fig 3). However, there is a preference for coupling to Gαq11, with little or no coupling with Gα12/13.^97, 98^ Gαq/11 signaling activates phospholipase C (PLC), which drives the formation of diacyl-glycerol (DAG) and inositol 1,4,5-triphosphate (IP3). IP3 activates calcium channels in the endoplasmic reticulum, leading to calcium release and DAG activates protein kinase C (PKC). Specifically, Gαs activation induces adenylyl cyclase activity, which results in cyclic AMP (cAMP) formation, leading to the activation of protein kinase A (PKA). Mrgprd is the only Mrgpr known to employ Gαs.^97^ Further, many Mrgprs employ the inhibitory Gαi pathway.^99, 100^ This pathway impedes adenylyl cyclase, suppressing cAMP production and consequently driving the inhibition of the stimulatory Gαs pathway. In addition, some Mrgprs, like MrgprA3, MrgprC11, and MRGPRX1, exhibit the utilization of Gαγ-signaling. Like various Gα signaling pathways, Gβγ can also activate PLC, PKC, and PKA. MRGPRX2 has been shown to activate to almost all G-coupled proteins but couples strongly to Gαq^88, 98^, as well as Gαi.^98, 101^

In addition to G-protein mediated signaling, ligand binding to GPCR also induces recruitment of adaptor β-arrestin proteins.^102^ Initially characterized for their role in GPCR desensitization and downregulation, β-arrestins can also initiate signaling events, resulting in unique biological outcomes.^103^ GPCR agonists, termed balanced agonists, can trigger both calcium and β-arrestin pathways. However, some GPCR agonists can selectively trigger G proteins or β-arrestins and are known respectively as G protein-biased and β-arrestin-biased agonists. Like other GPCRs, signaling downstream of Mrgprs can be different depending on the nature of the agonist. Research from Ali and his team has shed light on the activation of MRGPRX2, revealing two distinct activation models that depend on the ligand involved. For example, AG-30/5C, icatibant, codeine, C48/80 and the antimicrobial peptide LL-37 induced calcium mobilization and degranulation through MRGPRX2 via a G protein-dependent pathway; their impact on β-arrestin recruitment varies significantly. Notably, unlike AG-30/5C and LL-37, codeine and C48/80 induce MRGPRX2 internalization through β-arrestin^20, 26, 104^, which may function to restrain hyperactivation of MRGPRX2.^105^ Downstream of these pathways, MRGPRX2-mediated degranulation in response to C48/80 and substance P requires ERK and PI3K in primary human mast cells.^106, 107^ Similarly, C48/80- and LL-37-induced chemokines also appear to rely on ERK activation in the LAD2 human mast cell line.^108^ Recently, a non-synonymous polymorphism (N62S) in *MRGPRX2* was identified in ulcerative colitis (UC) patients.^109^ The serine (S) allele was shown to protect against UC. In response to various MRGPRX2 ligands, including PAMP12, the 62S variant was shown to induce significantly more β-arrestin recruitment and ERK phosphorylation but less inositol trisphosphate accumulation than the wildtype asparagine (N) allele (62N). Adrenomedullin, the PAMP12 precursor, is increased in inflamed UC tissue and it is speculated that this could drive pathology through the wildtype MRGPRX2. However, patients harboring the N62S variant, possibly due to increased β-arrestin-mediated MRGPRX2 desensitization, could be protected from aberrant activation by endogenous ligands.

Stimulation through FcεRI and MRGPRX2 are fundamentally distinct modes of mast cell activation, however they both appear to converge to the downstream activation of lysyl-tRNA synthetase (LysRS), a component of the translation machinery and a regulator of the transcription factor MITF (Microphthalmia associated-transcription factor). MITF is a well-known driver of melanocyte differentiation, but it also has a key role in driving mast cell identity. In response to FcεRI or MRGPRX2-dependent (via substance P) stimulation, the LysRS-MITF pathway drives genes that characterize mast cell identity and function like *Kit* and *Mmcp6* as well as promote degranulation.^107, 110-112^ Although there may only be a partial signaling overlap between these two-mast cell activating modalities since blockade of this pathway only partially impaired MRGPRX2 activation.^107^ Further, MRGPRX4 has been shown to associate with receptor activating-modifying protein-2 (RAMP-2), but not other members of the RAMP family.^113^ RAMP-2 reduces the surface expression of MRGPRX4 (Fig. 3), as well as inhibits basal and agonist-induced signaling driven by the MRGPRX4 ligands nateglinide^114^ and bile acids.^115, 116^

Although the various MRGPRs may use similar downstream signaling effectors, we know that MRGPRs exhibit responsiveness to a wide range of structurally distinct agonists, encompassing both small molecules and large peptides. This suggested the presence of distinct extracellular ligand-binding sites within these GPCRs. This was confirmed by CryoEM 3D structural studies of MRGPRX1, MRGPRX2, MRGPRX4 and MRGPRD that have demonstrated diverse residue composition and charge distribution in their extracellular binding sites.^98, 100, 117-119^ This is well illustrated when comparing MRGPRX2 and MRGPRX4. The primary binding pocket between these two MRGPRs was found to be quite different. The negatively charged binding site of MRGPRX2 explains why many of its agonists are positively charged. Conversely, MRGPRX4 does not have a negatively charged binding pocket and has several basic residues that confer a positive charge and a preference for negatively charged ligands like bile acids. Lastly, due to the lack of selective agonists for MRGPRX3, MRGPRE, MRGPRF and MRGPRG, the downstream signaling effectors to these are unknown.

### Targeting Mrgprs in the clinic

Various clinical trials to block MRGPRs are underway (Table 1). MRGPRX4 is being targeted as it drives itch in response to pathological levels of bile acids and heme metabolites^115, 116^, as seen in patients with chronic liver and kidney diseases. Also, the blockade of MRGPRX2 is being studied for skin diseases where mast cell dysregulation is thought to play a pathological role. Here, we will discuss the human data supporting these studies.

In AD patients, nerve fibers show positivity for substance P are elevated as compared to controls.^120^ Further, another study showed upregulation of *TAC1* (the gene encoding SP) in itchy skin, that positively correlated with itch intensity in AD patients.^121^ Also, the number of mast cells and production of PAMP12 by keratinocytes was enhanced in patients with ACD (n=5) compared to controls (n=5).^49^ Urticaria, another common and heterogeneous skin condition characterized by itching, hives, and inflammation, is also thought to be driven by aberrant mast cell activity. ^122^ While acute urticaria (≤ 6 weeks) has identifiable triggers (food, drugs), chronic urticaria or CU (> 6 weeks-1 year) can be both inducible and spontaneous with unknown etiology.^123^ Degranulation of mast cells releases inflammatory mediators, including histamine, that result in sensory nerve activation, vasodilatation, plasma extravasation, and cellular recruitment, and these sequelae underlie the development of key features of urticaria like hives, itch, and angioedema.^122^ In a small cohort of CU patients, staining of skin sections showed that in severe CU patients (n=9), the total number of mast cells was not increased compared to nonatopic controls (n=13). However, the frequency of MRGPRX2+ mast cells was higher (47.0% ± 6.9%) as compared to controls (21.6% ± 7.8%).^124^ Further, eosinophils, which also accumulate in CU patients, are thought to play a role via the production of major basic protein and eosinophil peroxidase, which were shown to activate mast cells to release histamine in an MRGPRX2-dependent manner in cultured skin mast cells.^124^ Further, in a cohort of mild CU patients (n=10), it was shown that the skin test response, as measured by the mean wheal diameter to two MRGPRX2 activating drugs, atracurium and icatibant, but not histamine, was significantly higher compared to controls (n=10).^125^ These 2 studies, albeit with a small number of patients, show promise for targeting MRGPRX2 in CU patients. In addition to having greater sensitivities to drugs known to activate MRGPRX2, some CU patients show elevated levels of the MRGPRX2 agonist substance P.^126, 127^

While Mrgprb2 and MRGPRX2 expression in mouse and human mast cells respectively shows a dichotomy between connective tissue and mucosal sites^15^, there exists a broad diversity of mast cell states in humans, as recently shown.^16, 128^ Therefore, the relative expression of MRGPRX2 in a continuum of mast cell states may be varied. This complexity could dictate the efficacy of the MRGPRX2 antagonists currently in the clinic.

## Concluding remarks

So far, we know that Mrgprs are expressed by sensory neurons, mast cells, neutrophils, and dendritic cells, where they regulate their function in various scenarios, including infection, allergy, and somatosensation. Several essential aspects of their biology remain unknown: 1) what drives the expression of Mrgprs on immune cells in a restricted manner? Is their surface expression modulated during inflammation or by disease-defining modifiers like environmental exposures, nutritional states, biome composition? 2) The genes encoding Mgrprs are highly polymorphic, and the function of these mutations on immune cell function and disease is largely unknown, 3) What is the role of immune-expressed Mrgprs in developing immune cell lineages and 4) It is important to appreciate that Mrgprs can function independently of their ligands, as they exhibit elevated basal activity.^129^ What is the functional consequence of this basal activity?

We anticipate that our knowledge of the immune functions of Mrgprs is not only going to expand but will also lead to the design of new clinical trials and the development of novel future therapeutics.

## Figures and Tables

**Table 1:** Drug development targeting MRGPRs

Target	Company	Disease	Phase	Comment
**MRGPRX2**	Escient Pharmaceuticals	AD and CU	Phase 1 completed Phase 2 ongoing for CU (NCT6077773) Phase 2 ongoing for AD (NCT06144424)	Safety trials found to be safe and well-tolerated
**MRGPRX2**	Evommune	Urticaria Interstitial Cystitis	Preclinical	N.A.
**MRGPRX4**	Escient Pharmaceuticals	Cholestatic/Uremic Pruritus	Phase 1 completed (NCT04510090) Phase 2 ongoing (NCT05525520)	Safety trials found to be safe and well-tolerated

## Box

The Mas-related G protein-coupled receptor (Mrgpr) family of orphan GPRCs are crucial for somatosensation, but more recently they have been shown to substantially impact immune cell function. Gour and Dong review the roles of Mrgprs expressed on immune cells and how they govern immune and neuro-immune pathways underlying various physiological and pathological states.

## References

[1] Liu Q, Tang Z, Surdenikova L, Kim S, Patel KN, Kim A, Sensory neuron-specific GPCR Mrgprs are itch receptors mediating chloroquine-induced pruritus. Cell. 2009;139(7):1353–65. 20004959 10.1016/j.cell.2009.11.034 PMC2989405

[2] Dong X, Han S, Zylka MJ, Simon MI, Anderson DJ. A diverse family of GPCRs expressed in specific subsets of nociceptive sensory neurons. Cell. 2001;106(5):619–32. 11551509 10.1016/s0092-8674(01)00483-4

[3] Lembo PM, Grazzini E, Groblewski T, O'Donnell D, Roy MO, Zhang J, Proenkephalin A gene products activate a new family of sensory neuron--specific GPCRs. Nat Neurosci. 2002;5(3):201–9. 11850634 10.1038/nn815

[4] Solinski HJ, Gudermann T, Breit A. Pharmacology and signaling of MAS-related G protein-coupled receptors. Pharmacol Rev. 2014;66(3):570–97. 24867890 10.1124/pr.113.008425

[5] Tatemoto K, Nozaki Y, Tsuda R, Konno S, Tomura K, Furuno M, Immunoglobulin E-independent activation of mast cell is mediated by Mrg receptors. Biochem Biophys Res Commun. 2006;349(4):1322–8. 16979137 10.1016/j.bbrc.2006.08.177

[6] Metcalfe DD. Mast cells and mastocytosis. Blood. 2008;112(4):946–56. 18684881 10.1182/blood-2007-11-078097 PMC2515131

[7] Galli SJ, Tsai M. IgE and mast cells in allergic disease. Nat Med. 2012;18(5):693–704. 22561833 10.1038/nm.2755 PMC3597223

[8] Hagermark O, Hokfelt T, Pernow B. Flare and itch induced by substance P in human skin. J Invest Dermatol. 1978;71(4):233–5. 81243 10.1111/1523-1747.ep12515092

[9] Gibbs BF, Wierecky J, Welker P, Henz BM, Wolff HH, Grabbe J. Human skin mast cells rapidly release preformed and newly generated TNF-alpha and IL-8 following stimulation with anti-IgE and other secretagogues. Exp Dermatol. 2001;10(5):312–20. 11589728 10.1034/j.1600-0625.2001.100503.x

[10] Caulfield JP, el-Lati S, Thomas G, Church MK. Dissociated human foreskin mast cells degranulate in response to anti-IgE and substance P. Lab Invest. 1990;63(4):502–10. 1700194

[11] McNeil BD, Pundir P, Meeker S, Han L, Undem BJ, Kulka M, Identification of a mast-cell-specific receptor crucial for pseudo-allergic drug reactions. Nature. 2015;519(7542):237–41. 25517090 10.1038/nature14022 PMC4359082

[12] Gentek R, Ghigo C, Hoeffel G, Bulle MJ, Msallam R, Gautier G, Hemogenic Endothelial Fate Mapping Reveals Dual Developmental Origin of Mast Cells. Immunity. 2018;48(6):1160–71 e5. 29858009 10.1016/j.immuni.2018.04.025

[13] Li Z, Liu S, Xu J, Zhang X, Han D, Liu J, Adult Connective Tissue-Resident Mast Cells Originate from Late Erythro-Myeloid Progenitors. Immunity. 2018;49(4):640–53 e5. 30332630 10.1016/j.immuni.2018.09.023

[14] Serhan N, Basso L, Sibilano R, Petitfils C, Meixiong J, Bonnart C, House dust mites activate nociceptor-mast cell clusters to drive type 2 skin inflammation. Nat Immunol. 2019;20(11):1435–43. 31591569 10.1038/s41590-019-0493-z PMC6858877

[15] Plum T, Wang X, Rettel M, Krijgsveld J, Feyerabend TB, Rodewald HR. Human Mast Cell Proteome Reveals Unique Lineage, Putative Functions, and Structural Basis for Cell Ablation. Immunity. 2020;52(2):404–16 e5. 32049054 10.1016/j.immuni.2020.01.012

[16] Tauber M, Basso L, Martin J, Bostan L, Pinto MM, Thierry GR, Landscape of mast cell populations across organs in mice and humans. J Exp Med. 2023;220(10). 10.1084/jem.20230570 PMC10354537 37462672

[17] Mencarelli A, Gunawan M, Yong KSM, Bist P, Tan WWS, Tan SY, A humanized mouse model to study mast cells mediated cutaneous adverse drug reactions. J Leukoc Biol. 2020;107(5):797–807. 31922289 10.1002/JLB.3MA1219-210RR PMC7322799

[18] Al Hamwi G, Riedel YK, Clemens S, Namasivayam V, Thimm D, Muller CE. MAS-related G protein-coupled receptors X (MRGPRX): Orphan GPCRs with potential as targets for future drugs. Pharmacol Ther. 2022;238:108259. 35934214 10.1016/j.pharmthera.2022.108259

[19] Lansu K, Karpiak J, Liu J, Huang XP, McCorvy JD, Kroeze WK, In silico design of novel probes for the atypical opioid receptor MRGPRX2. Nat Chem Biol. 2017;13(5):529–36. 28288109 10.1038/nchembio.2334 PMC5391270

[20] Babina M, Wang Z, Roy S, Guhl S, Franke K, Artuc M, MRGPRX2 Is the Codeine Receptor of Human Skin Mast Cells: Desensitization through beta-Arrestin and Lack of Correlation with the FcepsilonRI Pathway. J Invest Dermatol. 2021;141(5):1286–96 e4. 33058860 10.1016/j.jid.2020.09.017 PMC8041898

[21] Azimi E, Reddy VB, Lerner EA. Brief communication: MRGPRX2, atopic dermatitis and red man syndrome. Itch (Phila). 2017;2(1):e5. 28367504 10.1097/itx.0000000000000005 PMC5375112

[22] Wong GW, Zhuo L, Kimata K, Lam BK, Satoh N, Stevens RL. Ancient origin of mast cells. Biochem Biophys Res Commun. 2014;451(2):314–8. 25094046 10.1016/j.bbrc.2014.07.124 PMC4145527

[23] Brown KL, Hancock RE. Cationic host defense (antimicrobial) peptides. Curr Opin Immunol. 2006;18(1):24–30. 16337365 10.1016/j.coi.2005.11.004

[24] Mookherjee N, Anderson MA, Haagsman HP, Davidson DJ. Antimicrobial host defence peptides: functions and clinical potential. Nat Rev Drug Discov. 2020;19(5):311–32. 32107480 10.1038/s41573-019-0058-8

[25] Durr UH, Sudheendra US, Ramamoorthy A. LL-37, the only human member of the cathelicidin family of antimicrobial peptides. Biochim Biophys Acta. 2006;1758(9):1408–25. 16716248 10.1016/j.bbamem.2006.03.030

[26] Subramanian H, Gupta K, Guo Q, Price R, Ali H. Mas-related gene X2 (MrgX2) is a novel G protein-coupled receptor for the antimicrobial peptide LL-37 in human mast cells: resistance to receptor phosphorylation, desensitization, and internalization. J Biol Chem. 2011;286(52):44739–49. 22069323 10.1074/jbc.M111.277152 PMC3247983

[27] Subramanian H, Gupta K, Lee D, Bayir AK, Ahn H, Ali H. beta-Defensins activate human mast cells via Mas-related gene X2. J Immunol. 2013;191(1):345–52. 23698749 10.4049/jimmunol.1300023 PMC3691353

[28] Piliponsky AM, Romani L. The contribution of mast cells to bacterial and fungal infection immunity. Immunol Rev. 2018;282(1):188–97. 29431211 10.1111/imr.12623 PMC5812373

[29] Marshall JS, Portales-Cervantes L, Leong E. Mast Cell Responses to Viruses and Pathogen Products. Int J Mol Sci. 2019;20(17). 10.3390/ijms20174241 PMC6747121 31480219

[30] Krystel-Whittemore M, Dileepan KN, Wood JG. Mast Cell: A Multi-Functional Master Cell. Front Immunol. 2015;6:620. 26779180 10.3389/fimmu.2015.00620 PMC4701915

[31] Di Nardo A, Vitiello A, Gallo RL. Cutting edge: mast cell antimicrobial activity is mediated by expression of cathelicidin antimicrobial peptide. J Immunol. 2003;170(5):2274–8. 12594247 10.4049/jimmunol.170.5.2274

[32] Abraham SN, St John AL. Mast cell-orchestrated immunity to pathogens. Nat Rev Immunol. 2010;10(6):440–52. 20498670 10.1038/nri2782 PMC4469150

[33] Arifuzzaman M, Mobley YR, Choi HW, Bist P, Salinas CA, Brown ZD, MRGPR-mediated activation of local mast cells clears cutaneous bacterial infection and protects against reinfection. Sci Adv. 2019;5(1):eaav0216. 30613778 10.1126/sciadv.aav0216 PMC6314830

[34] Rutherford ST, Bassler BL. Bacterial quorum sensing: its role in virulence and possibilities for its control. Cold Spring Harb Perspect Med. 2012;2(11). 10.1101/cshperspect.a012427 PMC3543102 23125205

[35] Pundir P, Liu R, Vasavda C, Serhan N, Limjunyawong N, Yee R, A Connective Tissue Mast-Cell-Specific Receptor Detects Bacterial Quorum-Sensing Molecules and Mediates Antibacterial Immunity. Cell Host Microbe. 2019;26(1):114–22 e8. 31278040 10.1016/j.chom.2019.06.003 PMC6649664

[36] Rainer BM, Kang S, Chien AL. Rosacea: Epidemiology, pathogenesis, and treatment. Dermatoendocrinol. 2017;9(1):e1361574. 29484096 10.1080/19381980.2017.1361574 PMC5821167

[37] Daou H, Paradiso M, Hennessy K, Seminario-Vidal L. Rosacea and the Microbiome: A Systematic Review. Dermatol Ther (Heidelb). 2021;11(1):1–12. 33170492 10.1007/s13555-020-00460-1 PMC7859152

[38] Muto Y, Wang Z, Vanderberghe M, Two A, Gallo RL, Di Nardo A. Mast cells are key mediators of cathelicidin-initiated skin inflammation in rosacea. J Invest Dermatol. 2014;134(11):2728–36. 24844861 10.1038/jid.2014.222 PMC4199909

[39] Aroni K, Tsagroni E, Kavantzas N, Patsouris E, Ioannidis E. A study of the pathogenesis of rosacea: how angiogenesis and mast cells may participate in a complex multifactorial process. Arch Dermatol Res. 2008;300(3):125–31. 18071725 10.1007/s00403-007-0816-z

[40] Roy S, Alkanfari I, Chaki S, Ali H. Role of MrgprB2 in Rosacea-Like Inflammation in Mice: Modulation by beta-Arrestin 2. J Invest Dermatol. 2022;142(11):2988–97 e3. 35644498 10.1016/j.jid.2022.05.005 PMC9634617

[41] Li X, Yang H, Han Y, Yin S, Shen B, Wu Y, Tick peptides evoke itch by activating MrgprC11/MRGPRX1 to sensitize TRPV1 in pruriceptors. J Allergy Clin Immunol. 2021;147(6):2236–48 e16. 33358893 10.1016/j.jaci.2020.12.626

[42] Yosipovitch G, Rosen JD, Hashimoto T. Itch: From mechanism to (novel) therapeutic approaches. J Allergy Clin Immunol. 2018;142(5):1375–90. 30409247 10.1016/j.jaci.2018.09.005

[43] Shim WS, Tak MH, Lee MH, Kim M, Kim M, Koo JY, TRPV1 mediates histamine-induced itching via the activation of phospholipase A2 and 12-lipoxygenase. J Neurosci. 2007;27(9):2331–7. 17329430 10.1523/JNEUROSCI.4643-06.2007 PMC6673467

[44] Rosa AC, Fantozzi R. The role of histamine in neurogenic inflammation. Br J Pharmacol. 2013;170(1):38–45. 23734637 10.1111/bph.12266 PMC3764847

[45] Schmelz M, Petersen LJ. Neurogenic inflammation in human and rodent skin. News Physiol Sci. 2001;16:33–7. 11390944 10.1152/physiologyonline.2001.16.1.33

[46] Garibyan L, Rheingold CG, Lerner EA. Understanding the pathophysiology of itch. Dermatol Ther. 2013;26(2):84–91. 23551365 10.1111/dth.12025 PMC3696473

[47] Yu H, Usoskin D, Nagi SS, Hu Y, Kupari J, Bouchatta O, Single-Soma Deep RNA Sequencing of Human Dorsal Root Ganglion Neurons Reveals Novel Molecular and Cellular Mechanisms Underlying Somatosensation. bioRxiv. 2023:2023.03.17.533207.

[48] Usoskin D, Furlan A, Islam S, Abdo H, Lonnerberg P, Lou D, Unbiased classification of sensory neuron types by large-scale single-cell RNA sequencing. Nat Neurosci. 2015;18(1):145–53. 25420068 10.1038/nn.3881

[49] Meixiong J, Anderson M, Limjunyawong N, Sabbagh MF, Hu E, Mack MR, Activation of Mast-Cell-Expressed Mas-Related G-Protein-Coupled Receptors Drives Non-histaminergic Itch. Immunity. 2019;50(5):1163–71 e5. 31027996 10.1016/j.immuni.2019.03.013 PMC6531358

[50] Wang F, Trier AM, Li F, Kim S, Chen Z, Chai JN, A basophil-neuronal axis promotes itch. Cell. 2021;184(2):422–40 e17. 33450207 10.1016/j.cell.2020.12.033 PMC7878015

[51] Mack MR, Brestoff JR, Berrien-Elliott MM, Trier AM, Yang TB, McCullen M, Blood natural killer cell deficiency reveals an immunotherapy strategy for atopic dermatitis. Sci Transl Med. 2020;12(532). 10.1126/scitranslmed.aay1005 PMC7433875 32102931

[52] Kaplan DH, Igyarto BZ, Gaspari AA. Early immune events in the induction of allergic contact dermatitis. Nat Rev Immunol. 2012;12(2):114–24. 22240625 10.1038/nri3150 PMC3578582

[53] Neis MM, Peters B, Dreuw A, Wenzel J, Bieber T, Mauch C, Enhanced expression levels of IL-31 correlate with IL-4 and IL-13 in atopic and allergic contact dermatitis. J Allergy Clin Immunol. 2006;118(4):930–7. 17030248 10.1016/j.jaci.2006.07.015

[54] Steinhoff M, Neisius U, Ikoma A, Fartasch M, Heyer G, Skov PS, Proteinase-activated receptor-2 mediates itch: a novel pathway for pruritus in human skin. J Neurosci. 2003;23(15):6176–80. 12867500 10.1523/JNEUROSCI.23-15-06176.2003 PMC6740542

[55] Kempkes C, Buddenkotte J, Cevikbas F, Buhl T, Steinhoff M. Role of PAR-2 in Neuroimmune Communication and Itch. In: Carstens E, Akiyama T, editors. Itch: Mechanisms and Treatment. Frontiers in Neuroscience. Boca Raton (FL) 2014. 24829999

[56] Owen JL, Vakharia PP, Silverberg JI. The Role and Diagnosis of Allergic Contact Dermatitis in Patients with Atopic Dermatitis. Am J Clin Dermatol. 2018;19(3):293–302. 29305764 10.1007/s40257-017-0340-7 PMC5948135

[57] Thomsen SF. Atopic dermatitis: natural history, diagnosis, and treatment. ISRN Allergy. 2014;2014:354250. 25006501 10.1155/2014/354250 PMC4004110

[58] Brandt EB, Sivaprasad U. Th2 Cytokines and Atopic Dermatitis. J Clin Cell Immunol. 2011;2(3). 10.4172/2155-9899.1000110 PMC3189506 21994899

[59] Kawakami T, Ando T, Kimura M, Wilson BS, Kawakami Y. Mast cells in atopic dermatitis. Curr Opin Immunol. 2009;21(6):666–78. 19828304 10.1016/j.coi.2009.09.006 PMC2839879

[60] Paller AS, Kong HH, Seed P, Naik S, Scharschmidt TC, Gallo RL, The microbiome in patients with atopic dermatitis. J Allergy Clin Immunol. 2019;143(1):26–35. 30476499 10.1016/j.jaci.2018.11.015 PMC7163929

[61] Ogonowska P, Gilaberte Y, Baranska-Rybak W, Nakonieczna J. Colonization With Staphylococcus aureus in Atopic Dermatitis Patients: Attempts to Reveal the Unknown. Front Microbiol. 2020;11:567090. 33505363 10.3389/fmicb.2020.567090 PMC7830525

[62] Reddy VB, Lerner EA. Activation of mas-related G-protein-coupled receptors by the house dust mite cysteine protease Der p1 provides a new mechanism linking allergy and inflammation. J Biol Chem. 2017;292(42):17399–406. 28768771 10.1074/jbc.M117.787887 PMC5655516

[63] Snelgrove RJ, Gregory LG, Peiro T, Akthar S, Campbell GA, Walker SA, Alternaria-derived serine protease activity drives IL-33-mediated asthma exacerbations. J Allergy Clin Immunol. 2014;134(3):583–92 e6. 24636086 10.1016/j.jaci.2014.02.002 PMC4152000

[64] Lefrancais E, Duval A, Mirey E, Roga S, Espinosa E, Cayrol C, Central domain of IL-33 is cleaved by mast cell proteases for potent activation of group-2 innate lymphoid cells. Proc Natl Acad Sci U S A. 2014;111(43):15502–7. 25313073 10.1073/pnas.1410700111 PMC4217470

[65] Trier AM, Mack MR, Fredman A, Tamari M, Ver Heul AM, Zhao Y, IL-33 signaling in sensory neurons promotes dry skin itch. J Allergy Clin Immunol. 2022;149(4): 1473–80 e6. 34560104 10.1016/j.jaci.2021.09.014 PMC8934751

[66] Savinko T, Matikainen S, Saarialho-Kere U, Lehto M, Wang G, Lehtimaki S, IL-33 and ST2 in atopic dermatitis: expression profiles and modulation by triggering factors. J Invest Dermatol. 2012;132(5):1392–400. 22277940 10.1038/jid.2011.446

[67] Nakamura N, Tamagawa-Mineoka R, Yasuike R, Masuda K, Matsunaka H, Murakami Y, Stratum corneum interleukin-33 expressions correlate with the degree of lichenification and pruritus in atopic dermatitis lesions. Clin Immunol. 2019;201:1–3. 30772598 10.1016/j.clim.2019.02.006

[68] Li C, Maillet I, Mackowiak C, Viala C, Di Padova F, Li M, Experimental atopic dermatitis depends on IL-33R signaling via MyD88 in dendritic cells. Cell Death Dis. 2017;8(4):e2735. 28383552 10.1038/cddis.2017.90 PMC5477596

[69] Chen YL, Gutowska-Owsiak D, Hardman CS, Westmoreland M, MacKenzie T, Cifuentes L, Proof-of-concept clinical trial of etokimab shows a key role for IL-33 in atopic dermatitis pathogenesis. Sci Transl Med. 2019;11(515). 10.1126/scitranslmed.aax2945 31645451

[70] Keith YH, Egawa G, Honda T, Kabashima K. Mast cells in type 2 skin inflammation: Maintenance and function. Eur J Immunol. 2023;53(8):e2250359. 36933268 10.1002/eji.202250359

[71] Cavanaugh DJ, Lee H, Lo L, Shields SD, Zylka MJ, Basbaum AI, Distinct subsets of unmyelinated primary sensory fibers mediate behavioral responses to noxious thermal and mechanical stimuli. Proc Natl Acad Sci U S A. 2009;106(22):9075–80. 19451647 10.1073/pnas.0901507106 PMC2683885

[72] Zhang S, Edwards TN, Chaudhri VK, Wu J, Cohen JA, Hirai T, Nonpeptidergic neurons suppress mast cells via glutamate to maintain skin homeostasis. Cell. 2021;184(8):2151–66 e16. 33765440 10.1016/j.cell.2021.03.002 PMC8052305

[73] Chiu IM, Heesters BA, Ghasemlou N, Von Hehn CA, Zhao F, Tran J, Bacteria activate sensory neurons that modulate pain and inflammation. Nature. 2013;501 (7465):52–7. 23965627 10.1038/nature12479 PMC3773968

[74] Lin JJ, Du Y, Cai WK, Kuang R, Chang T, Zhang Z, Toll-like receptor 4 signaling in neurons of trigeminal ganglion contributes to nociception induced by acute pulpitis in rats. Sci Rep. 2015;5:12549. 26224622 10.1038/srep12549 PMC4519790

[75] Meseguer V, Alpizar YA, Luis E, Tajada S, Denlinger B, Fajardo O, TRPA1 channels mediate acute neurogenic inflammation and pain produced by bacterial endotoxins. Nat Commun. 2014;5:3125. 24445575 10.1038/ncomms4125 PMC3905718

[76] Segelcke D, Pradier B, Reichl S, Schafer LC, Pogatzki-Zahn EM. Investigating the Role of Ly6G(+) Neutrophils in Incisional and Inflammatory Pain by Multidimensional Pain-Related Behavioral Assessments: Bridging the Translational Gap. Front Pain Res (Lausanne). 2021;2:735838. 35295496 10.3389/fpain.2021.735838 PMC8915677

[77] Sahbaie P, Li X, Shi X, Clark JD. Roles of Gr-1+ leukocytes in postincisional nociceptive sensitization and inflammation. Anesthesiology. 2012;117(3):602–12. 22820848 10.1097/ALN.0b013e3182655f9f PMC3427475

[78] Green DP, Limjunyawong N, Gour N, Pundir P, Dong X. A Mast-Cell-Specific Receptor Mediates Neurogenic Inflammation and Pain. Neuron. 2019;101(3):412–20 e3. 30686732 10.1016/j.neuron.2019.01.012 PMC6462816

[79] Sbei S, Moncrief T, Limjunyawong N, Zeng Y, Green DP. PACAP activates MRGPRX2 on meningeal mast cells to drive migraine-like pain. Sci Rep. 2023;13(1):12302. 37516794 10.1038/s41598-023-39571-y PMC10387048

[80] Schytz HW, Birk S, Wienecke T, Kruuse C, Olesen J, Ashina M. PACAP38 induces migraine-like attacks in patients with migraine without aura. Brain. 2009;132(Pt 1):16–25. 19052139 10.1093/brain/awn307

[81] Bhatt DK, Gupta S, Olesen J, Jansen-Olesen I. PACAP-38 infusion causes sustained vasodilation of the middle meningeal artery in the rat: possible involvement of mast cells. Cephalalgia. 2014;34(11):877–86. 24563332 10.1177/0333102414523846

[82] Navratilova E, Porreca F. Substance P and Inflammatory Pain: Getting It Wrong and Right Simultaneously. Neuron. 2019;101(3):353–5. 30731054 10.1016/j.neuron.2019.01.034

[83] Ashina M, Dolezil D, Bonner JH, Zhou L, Klatt J, Picard H, A phase 2, randomized, double-blind, placebo-controlled trial of AMG 301, a pituitary adenylate cyclase-activating polypeptide PAC1 receptor monoclonal antibody for migraine prevention. Cephalalgia. 2021;41(1):33–44. 33231489 10.1177/0333102420970889 PMC7786389

[84] Son H, Zhang Y, Shannonhouse J, Ishida H, Gomez R, Kim YS. Mast-cell-specific receptor mediates alcohol-withdrawal-associated headache in male mice. Neuron. 2023. 10.1016/j.neuron.2023.09.039 PMC10843090 37909038

[85] Lariviere WR, Melzack R. The role of corticotropin-releasing factor in pain and analgesia. Pain. 2000;84(1):1–12. 10601667 10.1016/S0304-3959(99)00193-1

[86] Wedi B, Gehring M, Kapp A. The pseudoallergen receptor MRGPRX2 on peripheral blood basophils and eosinophils: Expression and function. Allergy. 2020;75(9):2229–42. 32003863 10.1111/all.14213

[87] Toscano A, Elst J, Van Gasse AL, Beyens M, van der Poorten ML, Bridts CH, Mas-related G protein-coupled receptor MRGPRX2 in human basophils: Expression and functional studies. Front Immunol. 2022;13:1026304. 36726977 10.3389/fimmu.2022.1026304 PMC9885256

[88] Robas N, Mead E, Fidock M. MrgX2 is a high potency cortistatin receptor expressed in dorsal root ganglion. J Biol Chem. 2003;278(45):44400–4. 12915402 10.1074/jbc.M302456200

[89] Meixiong J, Vasavda C, Green D, Zheng Q, Qi L, Kwatra SG, Identification of a bilirubin receptor that may mediate a component of cholestatic itch. Elife. 2019;8. 10.7554/eLife.44116 PMC6368403 30657454

[90] Azimi E, Reddy VB, Pereira PJS, Talbot S, Woolf CJ, Lerner EA. Substance P activates Mas-related G protein-coupled receptors to induce itch. J Allergy Clin Immunol. 2017;140(2):447–53 e3. 28219706 10.1016/j.jaci.2016.12.980 PMC5546940

[91] Perner C, Flayer CH, Zhu X, Aderhold PA, Dewan ZNA, Voisin T, Substance P Release by Sensory Neurons Triggers Dendritic Cell Migration and Initiates the Type-2 Immune Response to Allergens. Immunity. 2020;53(5):1063–77 e7. 33098765 10.1016/j.immuni.2020.10.001 PMC7677179

[92] Seaf M, Ben-Zimra M, Mankuta D, Dayan N, Levi-Schaffer F. Papain Activates Human Mast Cells to Release Proinflammatory Mediators via its Enzymatic Activity. J Invest Dermatol. 2016;136(7):1523–5. 27060447 10.1016/j.jid.2016.03.030

[93] Ericson JA, Duffau P, Yasuda K, Ortiz-Lopez A, Rothamel K, Rifkin IR, Gene expression during the generation and activation of mouse neutrophils: implication of novel functional and regulatory pathways. PLoS One. 2014;9(10):e108553. 25279834 10.1371/journal.pone.0108553 PMC4184787

[94] Dong X, Limjunyawong N, Sypek EI, Wang G, Ortines RV, Youn C, Keratinocyte-derived defensins activate neutrophil-specific receptors Mrgpra2a/b to prevent skin dysbiosis and bacterial infection. Immunity. 2022;55(9):1645–62 e7. 35882236 10.1016/j.immuni.2022.06.021 PMC9474599

[95] Tseng PY, Hoon MA. Specific beta-Defensins Stimulate Pruritus through Activation of Sensory Neurons. J Invest Dermatol. 2022;142(3 Pt A):594–602. 34480893 10.1016/j.jid.2021.07.178 PMC9549968

[96] Weis WI, Kobilka BK. The Molecular Basis of G Protein-Coupled Receptor Activation. Annu Rev Biochem. 2018;87:897–919. 29925258 10.1146/annurev-biochem-060614-033910 PMC6535337

[97] Steele HR, Han L. The signaling pathway and polymorphisms of Mrgprs. Neurosci Lett. 2021;744:135562. 33388356 10.1016/j.neulet.2020.135562 PMC8785421

[98] Cao C, Kang HJ, Singh I, Chen H, Zhang C, Ye W, Structure, function and pharmacology of human itch GPCRs. Nature. 2021;600(7887):170–5. 34789874 10.1038/s41586-021-04126-6 PMC9150435

[99] Ogasawara H, Noguchi M. Therapeutic Potential of MRGPRX2 Inhibitors on Mast Cells. Cells. 2021;10(11). 10.3390/cells10112906 PMC8616451 34831128

[100] Guo L, Zhang Y, Fang G, Tie L, Zhuang Y, Xue C, Ligand recognition and G protein coupling of the human itch receptor MRGPRX1. Nat Commun. 2023;14(1):5004. 37591889 10.1038/s41467-023-40705-z PMC10435460

[101] Chompunud Na Ayudhya C, Roy S, Alkanfari I, Ganguly A, Ali H. Identification of Gain and Loss of Function Missense Variants in MRGPRX2's Transmembrane and Intracellular Domains for Mast Cell Activation by Substance P. Int J Mol Sci. 2019;20(21). 10.3390/ijms20215247 PMC6862462 31652731

[102] Gurevich VV, Gurevich EV. GPCR Signaling Regulation: The Role of GRKs and Arrestins. Front Pharmacol. 2019;10:125. 30837883 10.3389/fphar.2019.00125 PMC6389790

[103] Jean-Charles PY, Kaur S, Shenoy SK. G Protein-Coupled Receptor Signaling Through beta-Arrestin-Dependent Mechanisms. J Cardiovasc Pharmacol. 2017;70(3):142–58. 28328745 10.1097/FJC.0000000000000482 PMC5591062

[104] Roy S, Ganguly A, Haque M, Ali H. Angiogenic Host Defense Peptide AG-30/5C and Bradykinin B(2) Receptor Antagonist Icatibant Are G Protein Biased Agonists for MRGPRX2 in Mast Cells. J Immunol. 2019;202(4):1229–38. 30651343 10.4049/jimmunol.1801227 PMC6369923

[105] Wang Z, Li Z, Bal G, Franke K, Zuberbier T, Babina M. beta-arrestin-1 and beta-arrestin-2 Restrain MRGPRX2-Triggered Degranulation and ERK1/2 Activation in Human Skin Mast Cells. Front Allergy. 2022;3:930233. 35910860 10.3389/falgy.2022.930233 PMC9337275

[106] Wang Z, Franke K, Bal G, Li Z, Zuberbier T, Babina M. MRGPRX2-Mediated Degranulation of Human Skin Mast Cells Requires the Operation of G(alphai), G(alphaq), Ca++ Channels, ERK1/2 and PI3K-Interconnection between Early and Late Signaling. Cells. 2022;11(6). 10.3390/cells11060953 PMC8946553 35326404

[107] Guo Y, Olle L, Proano-Perez E, Aparicio C, Guerrero M, Munoz-Cano R, MRGPRX2 signaling involves the Lysyl-tRNA synthetase and MITF pathway. Front Immunol. 2023;14:1154108. 37234172 10.3389/fimmu.2023.1154108 PMC10206166

[108] Callahan BN, Kammala AK, Syed M, Yang C, Occhiuto CJ, Nellutla R, Osthole, a Natural Plant Derivative Inhibits MRGPRX2 Induced Mast Cell Responses. Front Immunol. 2020;11:703. 32391014 10.3389/fimmu.2020.00703 PMC7194083

[109] Chen E, Chuang LS, Giri M, Villaverde N, Hsu NY, Sabic K, Inflamed Ulcerative Colitis Regions Associated With MRGPRX2-Mediated Mast Cell Degranulation and Cell Activation Modules, Defining a New Therapeutic Target. Gastroenterology. 2021;160(5):1709–24. 33421512 10.1053/j.gastro.2020.12.076 PMC8494017

[110] Lee YN, Nechushtan H, Figov N, Razin E. The function of lysyl-tRNA synthetase and Ap4A as signaling regulators of MITF activity in FcepsilonRI-activated mast cells. Immunity. 2004;20(2):145–51. 14975237 10.1016/s1074-7613(04)00020-2

[111] Ofir-Birin Y, Fang P, Bennett SP, Zhang HM, Wang J, Rachmin I, Structural switch of lysyl-tRNA synthetase between translation and transcription. Mol Cell. 2013;49(1):30–42. 23159739 10.1016/j.molcel.2012.10.010 PMC3766370

[112] Proano-Perez E, Olle L, Guo Y, Aparicio C, Guerrero M, Munoz-Cano R, MITF Downregulation Induces Death in Human Mast Cell Leukemia Cells and Impairs IgE-Dependent Degranulation. Int J Mol Sci. 2023;24(4). 10.3390/ijms24043515 PMC9961600 36834926

[113] Kotliar IB, Ceraudo E, Kemelmakher-Liben K, Oren DA, Lorenzen E, Dodig-Crnkovic T, Itch receptor MRGPRX4 interacts with the receptor activity-modifying proteins. J Biol Chem. 2023;299(5):104664. 37003505 10.1016/j.jbc.2023.104664 PMC10165273

[114] Kroeze WK, Sassano MF, Huang XP, Lansu K, McCorvy JD, Giguere PM, PRESTO-Tango as an open-source resource for interrogation of the druggable human GPCRome. Nat Struct Mol Biol. 2015;22(5):362–9. 25895059 10.1038/nsmb.3014 PMC4424118

[115] Yu H, Zhao T, Liu S, Wu Q, Johnson O, Wu Z, MRGPRX4 is a bile acid receptor for human cholestatic itch. Elife. 2019;8. 10.7554/eLife.48431 PMC6773440 31500698

[116] Meixiong J, Vasavda C, Snyder SH, Dong X. MRGPRX4 is a G protein-coupled receptor activated by bile acids that may contribute to cholestatic pruritus. Proc Natl Acad Sci U S A. 2019;116(21):10525–30. 31068464 10.1073/pnas.1903316116 PMC6535009

[117] Suzuki S, Iida M, Hiroaki Y, Tanaka K, Kawamoto A, Kato T, Structural insight into the activation mechanism of MrgD with heterotrimeric Gi-protein revealed by cryo-EM. Commun Biol. 2022;5(1):707. 35840655 10.1038/s42003-022-03668-3 PMC9287403

[118] Liu Y, Cao C, Huang XP, Gumpper RH, Rachman MM, Shih SL, Ligand recognition and allosteric modulation of the human MRGPRX1 receptor. Nat Chem Biol. 2023;19(4):416–22. 36302898 10.1038/s41589-022-01173-6

[119] Yang F, Guo L, Li Y, Wang G, Wang J, Zhang C, Structure, function and pharmacology of human itch receptor complexes. Nature. 2021;600(7887): 164–9. 34789875 10.1038/s41586-021-04077-y

[120] Jarvikallio A, Harvima IT, Naukkarinen A. Mast cells, nerves and neuropeptides in atopic dermatitis and nummular eczema. Arch Dermatol Res. 2003;295(1):2–7. 12709813 10.1007/s00403-002-0378-z

[121] Nattkemper LA, Tey HL, Valdes-Rodriguez R, Lee H, Mollanazar NK, Albornoz C, The Genetics of Chronic Itch: Gene Expression in the Skin of Patients with Atopic Dermatitis and Psoriasis with Severe Itch. J Invest Dermatol. 2018;138(6):1311–7. 29317264 10.1016/j.jid.2017.12.029

[122] Kolkhir P, Gimenez-Arnau AM, Kulthanan K, Peter J, Metz M, Maurer M. Urticaria. Nat Rev Dis Primers. 2022;8(1):61. 36109590 10.1038/s41572-022-00389-z

[123] Radonjic-Hoesli S, Hofmeier KS, Micaletto S, Schmid-Grendelmeier P, Bircher A, Simon D. Urticaria and Angioedema: an Update on Classification and Pathogenesis. Clin Rev Allergy Immunol. 2018;54(1):88–101. 28748365 10.1007/s12016-017-8628-1

[124] Fujisawa D, Kashiwakura J, Kita H, Kikukawa Y, Fujitani Y, Sasaki-Sakamoto T, Expression of Mas-related gene X2 on mast cells is upregulated in the skin of patients with severe chronic urticaria. J Allergy Clin Immunol. 2014;134(3):622–33 e9. 24954276 10.1016/j.jaci.2014.05.004

[125] Shtessel M, Limjunyawong N, Oliver ET, Chichester K, Gao L, Dong X, MRGPRX2 Activation Causes Increased Skin Reactivity in Patients with Chronic Spontaneous Urticaria. J Invest Dermatol. 2021;141(3):678–81 e2. 32771471 10.1016/j.jid.2020.06.030 PMC11658616

[126] Tedeschi A, Lorini M, Asero R. No evidence of increased serum substance P levels in chronic urticaria patients with and without demonstrable circulating vasoactive factors. Clin Exp Dermatol. 2005;30(2):171–5. 15725248 10.1111/j.1365-2230.2005.01732.x

[127] Metz M, Krull C, Hawro T, Saluja R, Groffik A, Stanger C, Substance P is upregulated in the serum of patients with chronic spontaneous urticaria. J Invest Dermatol. 2014;134(11):2833–6. 24844859 10.1038/jid.2014.226

[128] Dwyer DF, Ordovas-Montanes J, Allon SJ, Buchheit KM, Vukovic M, Derakhshan T, Human airway mast cells proliferate and acquire distinct inflammation-driven phenotypes during type 2 inflammation. Sci Immunol. 2021;6(56). 10.1126/sciimmunol.abb7221 PMC8362933 33637594

[129] Zeghal M, Laroche G, Freitas JD, Wang R, Giguere PM. Profiling of basal and ligand-dependent GPCR activities by means of a polyvalent cell-based high-throughput platform. Nat Commun. 2023;14(1):3684. 37407564 10.1038/s41467-023-39132-x PMC10322906

